# Interstrand crosslinking oligonucleotides elucidate the effect of metal ions on the methylation status of repetitive DNA elements

**DOI:** 10.3389/fchem.2023.1122474

**Published:** 2023-01-13

**Authors:** Shan Liu, Kunihiko Morihiro, Fumika Takeuchi, Yufeng Li, Akimitsu Okamoto

**Affiliations:** ^1^ Department of Chemistry and Biotechnology, Graduate School of Engineering, The University of Tokyo, Tokyo, Japan; ^2^ The Key Laboratory of Molecular Oncology of Hebei Province, Tangshan People’s Hospital, Tangshan, Hebei, China

**Keywords:** crosslinking, DNA methyaltion, epigenetics, fluorescent *in situ* hybridization (FISH), metal ion

## Abstract

DNA methylation plays an important physiological function in cells, and environmental changes result in fluctuations in DNA methylation levels. Metal ions have become both environmental and health concerns, as they have the potential to disrupt the genomic DNA methylation status, even on specific sequences. In the current research, the methylation status of two typical repetitive DNA elements, i.e., long-interspersed nuclear element-1 (LINE-1) and alpha satellite (α-sat), was imaged and assessed using methylation-specific fluorescence *in situ* hybridization (MeFISH). This technique elucidated the effect of several metal ions on the methylation levels of repetitive DNA sequences. The upregulation and downregulation of the methylation levels of repetitive DNA elements by various metal ions were confirmed and depended on their concentration. This is the first example to investigate the effects of metal ions on DNA methylation in a sequence-specific manner.

## Introduction

DNA methylation is a well-studied epigenetic mark that occurs exclusively at C-5 in the pyrimidine ring of cytosine (5-methylcytosine, 5mC). DNA methylation typically occurs in CpG dinucleotides and is present in about 70% of promoters. Jones (2012) It has been demonstrated that either the maintenance or the disturbance of DNA methylation leads to the promotion or dysfunction of biological processes, including embryonic development, genomic instability, and carcinogenesis; Hoffmann and Schulz (2005) DNA methylation is introduced into the genome *via* the DNA methyltransferase (DNMT) enzyme family, which comprises DNMT1, DNMT3A, and DNMT3B. Viegas-Péquignot et al. (2004), Lyko (2018) Conversely, the oxidative demethylation pathway is mediated by the ten-eleven translocation (TET) enzyme family, generating oxidative relatives: 5-hydroxymethylcytosine (5hmC), 5-formylcytosine (5fC), and 5-carboxycytosine (5caC). Kohli and Zhang (2013).

In recent years, fluctuations in DNA methylation under environmental changes, such as organic pollutants and metals, have been widely reported. Ruiz-Hernandez et al. (2015) Among these environmental factors, metal ions, which are frequently used in industrial processes, have become both an environmental and health concern. Although trace amounts of some metals are essential for humans to maintain health and orchestrate physiological functions, excessive absorption would result in the generation of reactive oxygen species, leading to DNA damage, and finally, threatening human health. Rehman et al. (2018) Several metals, including iron (Fe), copper (Cu), cobalt (Co), nickel (Ni), aluminum (Al), and zinc (Zn), have been characterized as being carcinogenic or disease-inducing metals. Arita and Costa (2009), Kim et al. (2015), Chen et al. (2019) Moreover, some of these metals disturb the DNA methylation status in cells, even on specific sequences. Martinez-Zamudio and Ha (2011), Xiong et al. (2017), Martin and Fry (2018) However, an overview of the epigenetic effects of metal ions on specific DNA elements has not been provided.

To estimate the DNA methylation status under metal ion exposure, long-interspersed nuclear element-1 (LINE-1) from interspersed repetitive sequences was selected as the indicator of genomic methylation in the current research. LINE-1 is an active interspersed repetitive sequence that originated from a retrotransposon and has a unit length of about 6.5 kb. Skowronski et al. (1988); Swergold (1990) There are approximately 500,000 LINE-1 copies in the human genome, composing 17% of the human DNA. (Rodić and Burns, 2013) LINE-1 is composed of a 5′-untranslated region (5′-UTR), two open reading frames (ORF1 and ORF2), and a 3′-UTR containing a polyA tail. Suarez et al. (2018) These active LINE-1s utilize a “copy and paste” mechanism to insert themselves throughout the whole genome; moreover, their retrotransposition potentially disrupts the expression of neighboring genes; Wolff et al. (2010); Blaudin de Thé et al. (2018) It has also been demonstrated that the 5′-UTR harbors two promoters, the methylation level of which affects the retrotransposition activity of LINE-1. Thus, a growing number of researchers take LINE-1 as a DNA methylation indicator for the whole genome; Fustinoni et al. (2007); Cho et al. (2019) In turn, the methylation status of another element from tandem repetitive DNA, alpha satellite (α-sat), was estimated together with LINE-1. α-Sat is a primate-specific DNA element that is arranged in an array of tandemly repeated units, each of about 171 bp, making up to ∼10% of the genome. Schueler and Sullivan (2006) α-Sat is mainly located at the centromeric region and plays important roles in *de novo* centromere assembly and cell division. Schueler and Sullivan (2006) It has been reported that arrays of α-sat monomers contribute to centromeric polymorphism, potentially as a useful biomarker of chromosome specificity and inheritance. McNulty and Sullivan (2018) Furthermore, according to a previous report, the combination of the low methylation status of α-sat together with LINE-1 was associated with a shortened survival time in patients with advanced gastric cancer, Kim et al. (2019) which indicates its diagnostic potential in cancer research.

**FIGURE 1:**
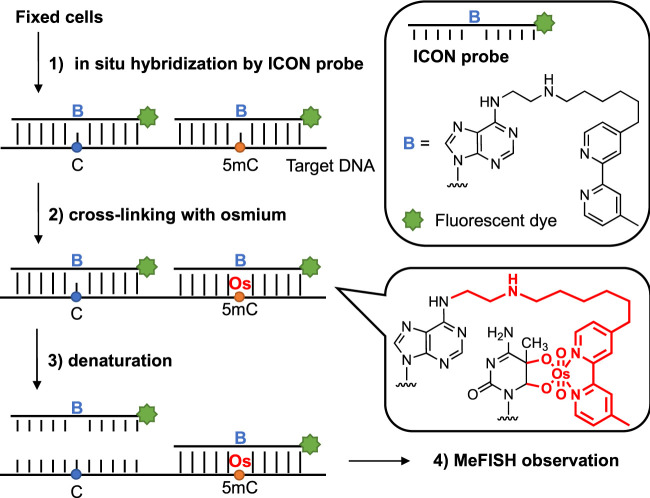
Sequence-specific 5 mC detection in DNA using ICON probes.

Various strategies have been established to date to analyze 5 mC in both DNA and RNA. For example, bisulfite conversion can discriminate 5 mC from unmethylated cytosine in which the 5 mC is protected, whereas cytosine is deaminated and converted to uracil, making it possible to map the methylation patterns of DNA. Frommer et al. (1992) Liquid chromatography-mass spectrometry (LC-MS) combined with chemical labeling can achieve high resolution, a low limit of detection, and quantitative analysis of 5 mC using a trace amount of DNA; however, the sequence information can be lost. Yuan and Feng (2014) In recent years, the high-throughput DNA sequencing technology aimed at detecting a single site of 5 mC has also afforded remarkable advancements in this field; Flusberg et al. (2010); Ambardar et al. (2016); Rand et al. (2017) Among these strategies, we mainly focused on microscopic observation, with the exception of the use of anti-5mC antibodies; Coffigny et al. (1999) Thus, a novel method termed methylation-specific fluorescence *in situ* hybridization (MeFISH), which is based on the difference in reactivity between 5 mC and cytosine in the target DNA for interstrand complex formation with osmium and nucleic acids (ICON), was established by Sasaki and our groups (Figure 1). Li et al. (2013) In this method, fixed cells are subjected to *in situ* hybridization using ICON probes, and non-binding probes are subsequently washed out. The specimens are treated with osmium to achieve crosslinking with 5mC, and non-crosslinked probes are removed by denaturation. Then, MeFISH observation enables the estimation of the DNA methylation status at specific sites. Compared with the methods mentioned above, the DNA methylation patterns of specific sequences in individual cells could be visualized by MeFISH. Moreover, the multi-imaging of DNA methylation on specific sequences and the distribution of other modifications or important enzymes could be realized by applying MeFISH in conjunction with immunostaining. Shiura et al. (2014) Based on these advantages, in the present study, MeFISH was employed to estimate the effects of various metals on the DNA methylation status at the single-cell level, followed by statistical analysis. We estimated the effects of several metal ions on both LINE-1 and α-sat methylation. We found that these metals had different effects on the LINE-1 and α-sat methylation levels, depending on their concentration and exposure time.

## Results

### Design and synthesis of ICON probes for LINE-1 and α-sat

**FIGURE 2:**
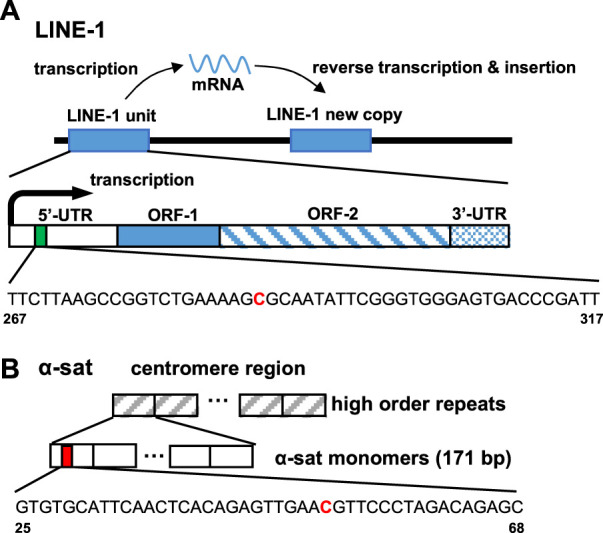
Target site of the ICON probe for **(A)** LINE-1 and **(B)** α-sat on human genomic DNA. The bold red capital letter C indicates the target cytosine.

The ability of ICON probes to discriminate 5 mC from normal cytosine in DNA is mainly based on the different reaction rates during osmium oxidation with bipyridine, because the methyl group on C-5 in 5 mC has been demonstrated to facilitate oxidation. Nomura et al. (2007) Thus, an ICON probe containing a bipyridine-attached adenine derivative at the position corresponding to the 5 mC allowed the sequence-specific detection of 5 mC in DNA. Tanaka et al. (2007) As shown in Table 1, the ICON probe for LINE-1 was designed according to the published sequence information, Waye and Willard (1986) followed by fluorescent labeling with 5′-FAM at the 5′-end. The ICON probes for LINE-1 mainly hybridize with the initial region of the LINE-1 5′-UTR, the GC percent of which is over 50%, including 29 methylatable CpG sites (Figure 2A). Baba et al. (2018) The ICON probe for α-sat followed the design of a previous application, Li et al. (2013) which was labeled with TAMRA at the 5′-end (Figure 2B). All bipyridine-attached adenine derivatives were introduced at the positions corresponding to the cytosine that possibly are methylated. In MeFISH, the samples are heated at 85°C for 10 min to fully denature genomic DNA for the following hybridization with ICON probes. Thus the probes can work for duplex DNA. Furthermore, RNA is digested with RNase A, which is specific for single-stranded RNA and commonly used in research to remove RNA from DNA. Thus the RNA being transcribed is undetectable by this method.

**TABLE 1:** Sequences of ICON probes for LINE-1 and α-sat. The bold capital letter B indicates the bipyridine-attached adenine.

Target	Sequence (5′→3′)
LINE-1	FAM-AATCGGGTCACTCCCACCCGAATATTGC**B**CTTTTCAGACCGGCTTAAGAA
α-sat	TAMRA-GCTCTGTCTAAGGGAAC**B**TTCAACTCTGTGAGTTGAATGCACAC

**FIGURE 3:**
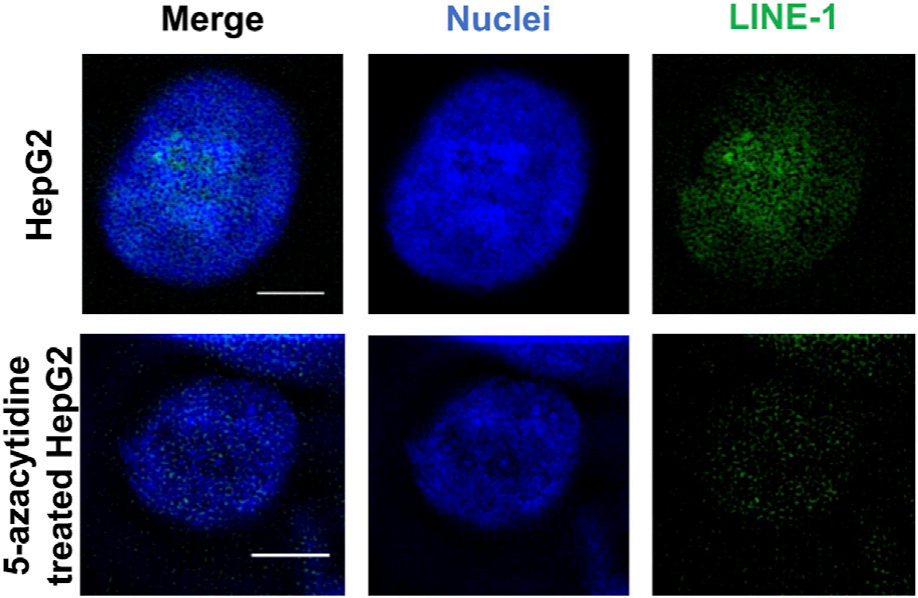
MeFISH images of LINE-1 (green) in normal HepG2 cells (blank control) and HepG2 cells treated with 1 μΜ 5-azacytidine, which inhibits DNA methylation. The nuclei were labelled by DAPI (blue). Scale bars, 5 μm.

We initially tested the function of the designed ICON probes on human liver cancer HepG2 cells, as an alternative model of the liver. HepG2 cells were subjected to MeFISH and the methylation status of LINE-1 was successfully imaged and analyzed (Figure 3). Conversely, the LINE-1 methylation level of HepG2 cells treated with 1 μΜ 5-azacytidine, which inhibits DNA methylation by trapping DNMT and inducing its degradation, Agrawal et al. (2018) was downregulated to ∼1/3 that detected in untreated HepG2 cells (Supplementary Figure S1). This initial investigation indicated that the designed ICON probe was able to image the changes in LINE-1 methylation status.

**FIGURE 4:**
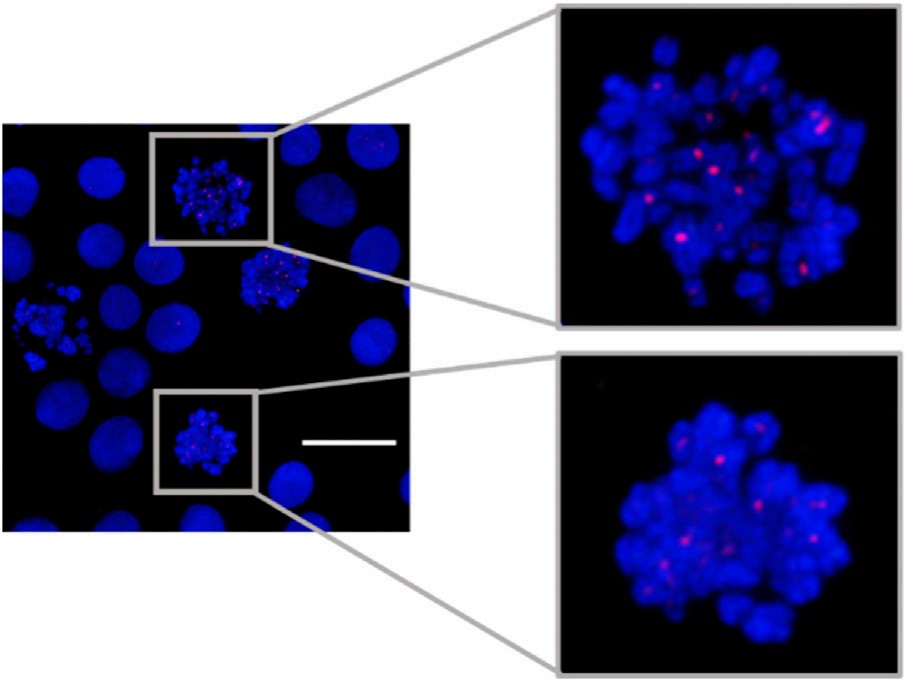
MeFISH images of α-sat (red) in HepG2 cells. The nuclei were labelled by DAPI (blue). Scale bar, 20 μm.

MeFISH for α-sat was also demonstrated (Figure 4). The ICON probe targeting α-sat was designed to bind to the centromere region and the microscopic images clearly show that TAMRA fluorescence is positioned at the desired locations. These results prompted us to use the designed ICON probes to investigate the effect of various metal ions on the repetitive DNA elements.

### Different metals had varying effects on LINE-1 and α-sat methylation

To estimate the effects of different metals on LINE-1 and α-sat methylation statuses at different concentrations, the following experiments were performed. Metal-treated HepG2 cells and untreated cells were prepared as described in the experimental section. The concentration of each metal ranged from 50 to 200 μM. The samples were fixed and stocked in Carnoy’s solution, followed by MeFISH, microscopic observation (Supplementary Figure S2), and statistical analysis, which were performed as described in the experimental section.

Here, we mainly focused on the effect of five metal ions: Cu(II), Co(II), Ni(II), Zn(II), and Al(III). Among them, Cu(II), Co(II), Ni(II), and Al(III) have been demonstrated to disturb the methylation status or retrotransposition activity of LINE-1. El-Sawy et al. (2005); Karimi et al. (2014) Although it has not been shown that Zn(II) is involved in DNA methylation directly, Zn(II) plays a vital role in the structure and function of many enzymes involved in the regulation of physiological processes, Kluska et al. (2018) which was also listed as the subjects of this research.

**FIGURE 5:**
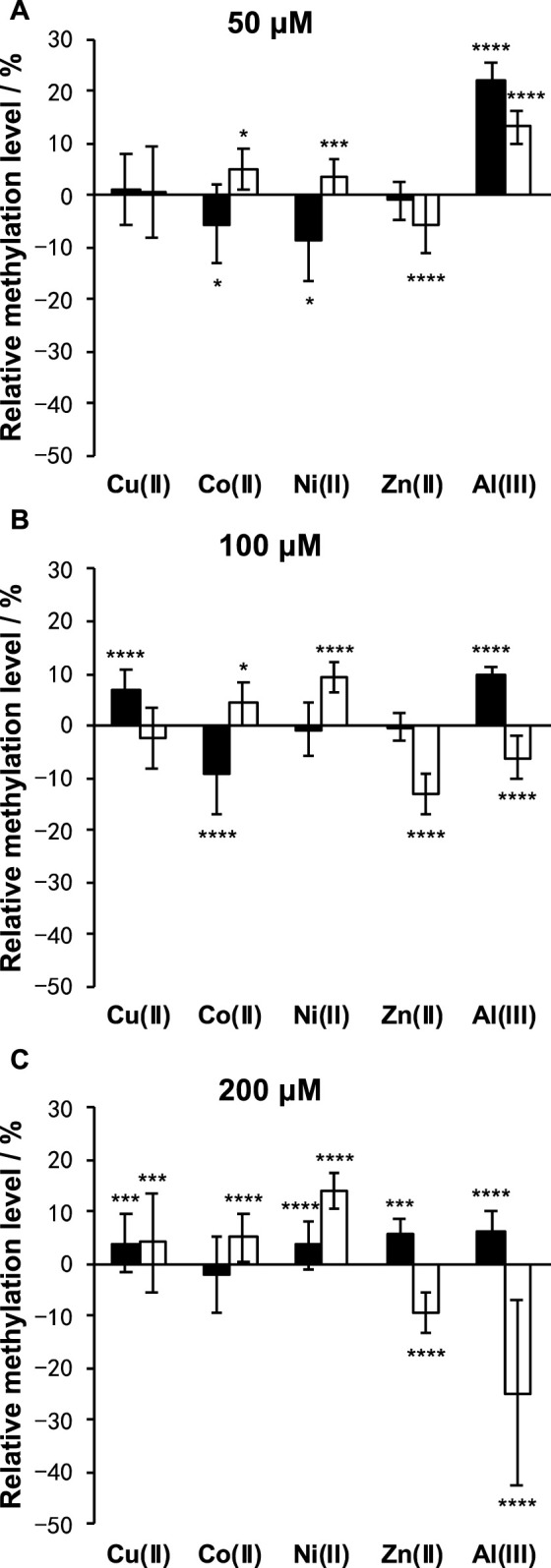
Effects of Cu(II), Co(II), Ni(II), Zn(II), and Al(III) on LINE-1 (black bar) and α-sat (white bar) methylation, at **(A)** 50 μM, **(B)** 100 μM, and **(C)** 200 μM. The differences between the treated and untreated group assays were evaluated after 1 day of exposure. The error bars represent standard errors. *p < .05, ***p < .005, and ****p < .001 by Mann-Whitney test.

**FIGURE 6:**
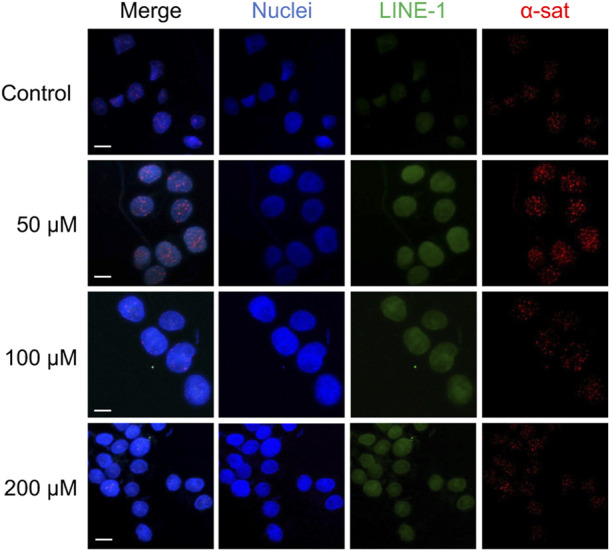
The microscopic images of HepG2 cells treated with various concentrations of Al (III). The nuclei were labelled by DAPI (blue). Scale bars, 10 μm.

As shown in Figure 5, Cu(II) and Co(II) had almost no effect on the methylation level of both LINE-1 and α-sat at each concentration (<10% changes). As the concentration of Ni(II) increased, the methylation level also gradually increased, especially for α-sat. Zn(II) triggered the downregulation of α-sat methylation in a concentration-independent manner. Al(III) significantly upregulated LINE-1 and α-sat methylation at 50 μM. However, the methylation level was gradually decreased as the metal concentration increased, and 200 μΜ Al(III) significantly downregulated α-sat methylation to −25% (The microscopic images are shown in Figure 6).

### Metal ions showed biphasic cytotoxicity in HepG2 cells

To estimate the effects of the metals on the physiological activity of cells, a cell viability assay was performed in triplicate for each metal at various concentrations, by estimating the reducing power of living cells using the PrestoBlue^®^ reagent. This assay revealed that a 24-h exposure to various concentrations of these metals in HepG2 cells produced a stimulative or reductive effect on cell viability (Supplementary Table S1). Moreover, it showed that in the presence of the metals selected for this research, the survival rate was over 80%, even at 200 μM, which was consistent with previously published reports. Yamamoto et al. (1998) With the exception of Zn(II), the assay also showed that these metals promoted cell proliferation at a certain concentration, indicating biphasic cytotoxicity in HepG2 cells.

## Discussion

**FIGURE 7:**
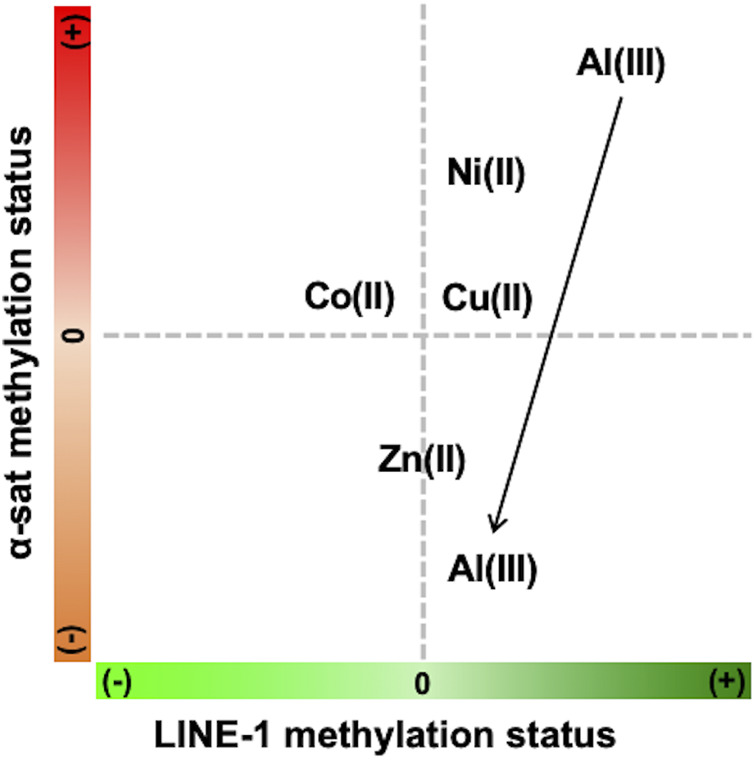
Summary of the concentration-dependent effects of various metals on LINE-1 and α-sat methylation status (+) refers to hypermethylation, and (−) refers to hypomethylation. The arrow indicates the increase of metal concentration.

As the concentration of Ni(II) increased, the methylation status of both LINE-1 and α-sat was upregulated. According to a previous report, Ni(II) inhibits TET-mediated 5 mC oxidative demethylation *via* high-affinity displacement of the cofactor Fe(II) in the catalytic domain of TET. Yin et al. (2017) It also showed that Ni(II) inhibited the DNA oxidative demethylation pathway in a dose-dependent way in both somatic cells and embryonic stem cells. Yin et al. (2018) This Ni(II) effect on epigenetics-related enzymes strongly supports the effects of Ni(II) on DNA methylation obtained by MeFISH. α-Sat plays an important role in chromosome stability and cell division. Recently, it has been demonstrated that Ni(II) exposure induces chromosome condensation by substituting Mg(II) on the DNA phosphate backbone, resulting in gene silencing as well as DNA hypermethylation. Sun et al. (2013) Here, we observed that as the concentration of Ni(II) increased, hypermethylation also increased in α-sat. As α-sat is located in centromeres, which exist in a condensed state, α-sat methylation potentially plays an important role in gene silencing under exposure to cytotoxic metals. The observed effects of Ni(II) on α-sat further concreted the reliability of MeFISH in the analysis of DNA methylation in specific sequences. Zn(II) exposure had an obvious effect only on α-sat methylation, and the DNA methylation status of α-sat was more sensitive to the exposure to various metal ions than was that of LINE-1. Exposure of HepG2 cells to Al(III) led to different effects on the methylation level of both LINE-1 and α-sat, depending on the concentration (Figure 7). According to a previous report, when the concentration of Al(III) was greater than 1.5 mM, the retrotransposition activity of LINE-1 in the HepG2 cell line was significantly upregulated, Karimi et al. (2014) indicating the decreased methylation status of LINE-1 under Al(III) exposure at high concentrations. Here, we found that the methylation status of LINE-1 gradually decreased as the concentration increased, referring to the more active retrotransposition of LINE-1. Recently, an Al(III)-induced neurotoxicity mouse model showed that a 42-day-long accumulation of Al(III) significantly downregulated the expression of DNMT3A in the hippocampus, but not in the cortex, thus highlighting the different effects of Al(III) in different tissues. Ikram et al. (2021) That report motivated us to assess whether Al(III) exposure regulates the expression of genes that are related to DNA methylation in the HepG2 cell line.

Although the mechanism *via* which these metals affect DNA methylation is complex and remains unknown, d-block metals, such as Cu(II), Co(II), Ni(II), and Zn(II), are located in the same row and share similar electron structures, thus facilitating diverse functions of proteins and their complexes. Kluska et al. (2018) Therefore, it is possible that these metals share the ability to substitute each other in the catalytic domains of enzymes, resulting in the activation/inactivation of certain enzymes, such as TET1 and the DNMT family, thus further disturbing the DNA methylation status of LINE-1 or α-sat. The previous study showed that in various clinical models with an assessment of age, sex, lifestyle, *etc.*, the methylation status of global LINE-1 significantly changed from less than 1% to over 30%, under the exposure of different metals; Ruiz-Hernandez et al. (2015) Besides, Byun and co-workers reported the effects of the airborne pollutant on LINE-1 subfamilies and showed that although the methylation difference was less than 2% in the considered position, the evolutionary age of LINE-1 was significantly changed, further related to human health and diseases; Byun et al. (2013) Taken together, it is possible that even though the methylation difference of certain repetitive DNA elements is small (in the current study, ranging from <10% to over 20%), it may lead to the instability of the whole genome.

## Conclusion

In conclusion, we applied MeFISH, which is a powerful tool used for site-selective DNA methylation analysis, to elucidate the effect of several metals on the methylation level of repetitive DNA elements. The epigenetic effects induced by Cu(II), Co(II), Ni(II), Zn(II), Al(III) and were estimated and discussed. We found that Ni(II) exposure upregulated LINE-1 methylation at high concentrations. In addition, Zn(II) decreased α-sat methylation in a concentration-independent manner. Moreover, Al(III) exposure had different effects on LINE-1 and α-sat, depending on the concentration. These metals affected the LINE-1 or α-sat methylation levels depending on their concentration and may become a piece of the puzzle to realize the association between the methylation status of retrotransposons and exposure to cytotoxic metals.

## Experimental

### Chemical labeling of ICON probes

ICON probes were bought from Gene Design Inc. (Japan) and contained a bipyridine-attached adenine derivative at the position corresponding to the methylated cytosine, and an amino group at their 5′end. The preparation of FAM-labeled ICON probes for LINE-1 and TAMRA-labeled ICON probes for α-sat was as follows. First, 7 μL of deionized water, 4 μL of 25 μg/μL ICON probe stock solution, and 250 μg of FAM or TAMRA in 14 μL of DMSO were added to 75 μL of labeling buffer (.1 M sodium tetraborate buffer, pH 8.5). The reaction mixture was vortexed for 1 min to increase the labeling efficiency, and then placed on a shaker oscillating at low speed overnight at 25°C. After the incubation, one-tenth volume of 3 M NaCl and two and a half volumes of cold absolute ethanol were added to the reaction mixture. The mixture was incubated at −20°C for 30 min, followed by centrifugation at 120,00× *g* for 40 min. After removing the supernatant carefully, the pellet was rinsed with cold 70% ethanol twice and dried briefly.

### Preparation and fixation of metal-exposed cells

The cell lines present in this study were obtained from RIKEN BRC CELL BANK. HepG2 cells were maintained in Dulbecco’s Modified Eagle’s Medium (DMEM, GIBCO) supplemented with 10% fetal bovine serum (FBS, Biowest), 100 U mL^−1^ penicillin, and 100 U mL^−1^ streptomycin (Nacalai Tesque) at 37°C in a 5% CO_2_ atmosphere. The metal exposure was performed as follows: 2 × 10^4^ cells were seeded in a 3.5-cm culture dish and first cultured with normal medium for 48 h; subsequently, they were washed with phosphate-buffered saline (PBS) and then treated with medium including FeCl_3_, AlCl_3_, CoCl_2_, CuSO_4_, NiCl_2_ and ZnCl_2_ (FUJIFILM Wako Pure Chemical Corporation), ranging from 20 to 200 μM, separately. After incubation for 24 h, the cells were fixed using the following steps. First, cells were digested with .25% trypsin/EDTA, followed by PBS washing. Then, the cells were gently suspended in a hypotonic solution (75 mM KCl) and allowed to stand for 8 min at room temperature. After the same volume of Carnoy’s solution (fixative solution, methanol/acetic acid (3/1, v/v)) was added, cells were mixed gently. After centrifugation at 1,500 rpm, the supernatant was discarded, and an equal volume of fresh Carnoy’s solution was added. This procedure was repeated twice. Fixed cells were stored in Carnoy’s solution at −20°C.

### MeFISH

The procedure used for MeFISH was as described previously, Li et al. (2013) with optimization for the current research. A drop of fixed cells was placed on a glass microscope slide and air dried. After digestion with 2 μg/mL RNase A (NIPPON GENE) and .02% pepsin (Nacalai Tesque), a hybridization mixture (4 μL) containing labeled probes (1.25 ng/μL each), 2× saline-sodium citrate (SSC) buffer, 0.5 mM EDTA, 10% dextran sulfate, and 25% formamide was applied onto the fixed cells, which were then sealed with a glass cover slip and rubber cement at room temperature. The slide was placed on a heating block at 85°C for 10 min, to denature the genomic DNA, and incubated in a moist chamber at room temperature overnight. The glass cover slip was removed by soaking in 2× SSC, and a post-hybridization wash was performed three times in 2× SSC at 37°C. A 15-μL crosslinking solution containing 12.5 mM K_2_OsO_4_ and 50 mM Tris-HCl (pH 7.7) with .5 mM EDTA and 1 M NaCl was added. The crosslinking was carried out in a moist atmosphere at 37°C. Non-crosslinked probes were removed by denaturation in 2× SSC with 70% formamide at 75°C for 5 min. Finally, the slide was washed with PBS and dehydrated. Fluorescence images of MeFISH were acquired by a Nikon A1 confocal laser microscope. It should be pointed out that the signal intensity of MeFISH can be affected by the chromatin structure and preparation conditions. Thus, a blank control was always prepared in parallel.

### Quantitative analysis of DNA methylation level by MeFISH

The signal intensity of each type of ICON probe was measured by ImageJ software (Wayne Rasband, NIH), from microscopic images. First, images obtained from all the detection channels were turned into grayscale for analysis. The area for analysis of each cell was confirmed *via* the detection channel of DAPI/nuclei. Then the signal intensity of each type of ICON probe was measured in the determined area, separately. Obtained results were reported as means ± standard errors of the mean, subjected to statistical analysis by MATLAB R2019b, using the Mann-Whitney test. The levels of significance were determined as *p < .05, **p < .01, ***p < .005, and ****p < .001.

### Cell viability assay

The cell viability assay was performed using the following procedure. 2 × 10^4^ HepG2 cells were seeded into each well of a 96-well plate. After a 48-h incubation, the medium was removed, and fresh medium containing various concentrations of Al(III), Co(II), Cu(II), Ni(II), and Zn(II) was added to each well. After a 24-h incubation, the medium was removed, and fresh medium containing 1× PrestoBlue™ Cell Viability Reagent (Thermo Fisher Scientific) was added to each well. After a 1-h incubation, the fluorescence intensity (λ_ex_ = 560 nm, λ_em_ = 590 nm) of each well was measured on a Bioteck Cytation5 plate reader. All treatment experiments were performed in triplicate, and the relative cell viability (%) was expressed as a percentage relative to the untreated control cells.

## Data Availability

The raw data supporting the conclusions of this article will be made available by the authors, without undue reservation.
