# Supplementary material for: Interstrand crosslinking oligonucleotides elucidate the effect of metal ions on the methylation status of repetitive DNA elements
Source: Front Chem. 2023 Jan 13;11:1122474. doi: 10.3389/fchem.2023.1122474 (PMC9881727; doi:10.3389/fchem.2023.1122474)

Supplementary Material

Interstrand Crosslinking Oligonucleotides Elucidate the Effect of Metal Ions on the Methylation Status of Repetitive DNA Elements

Shan Liu, Kunihiko Morihiro, Fumika Takeuchi, Yufeng Li, Akimitsu Okamoto*

*** Correspondence:** Akimitsu Okamoto: okamoto@chembio.t.u-tokyo.ac.jp

Table of contents

Page S2 **Supplementary Figure 1.** Quantitative analysis of change in LINE-1 methylation levels by 5-azacytidine treatment.

Page S3 **Supplementary Figure 2.** MeFISH images of LINE-1 (green) and α-sat (red) from HepG2 cells treated with Cu(II), Ni(II), Fe(III), Al(III), Co(II) and Zn(II), ranging from 50 μM to 200 μM.

Page S6 **Supplementary Table 1.** Cell viability assay.

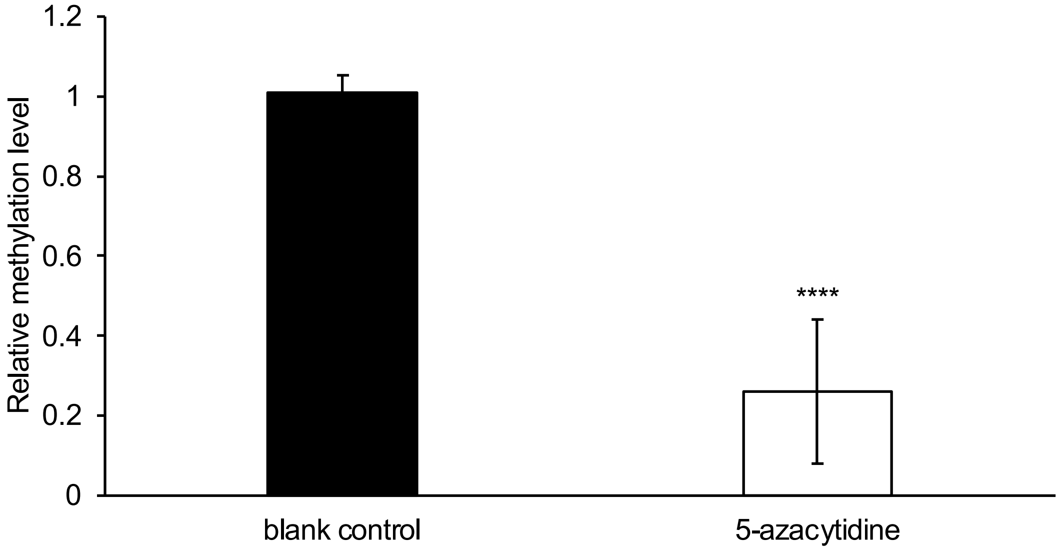

Supplementary Figure 1. Quantitative analysis of change in LINE-1 methylation levels by 5-azacytidine treatment. The error bars represent standard errors. ****$P < 0.001$ by Mann-Whitney test.

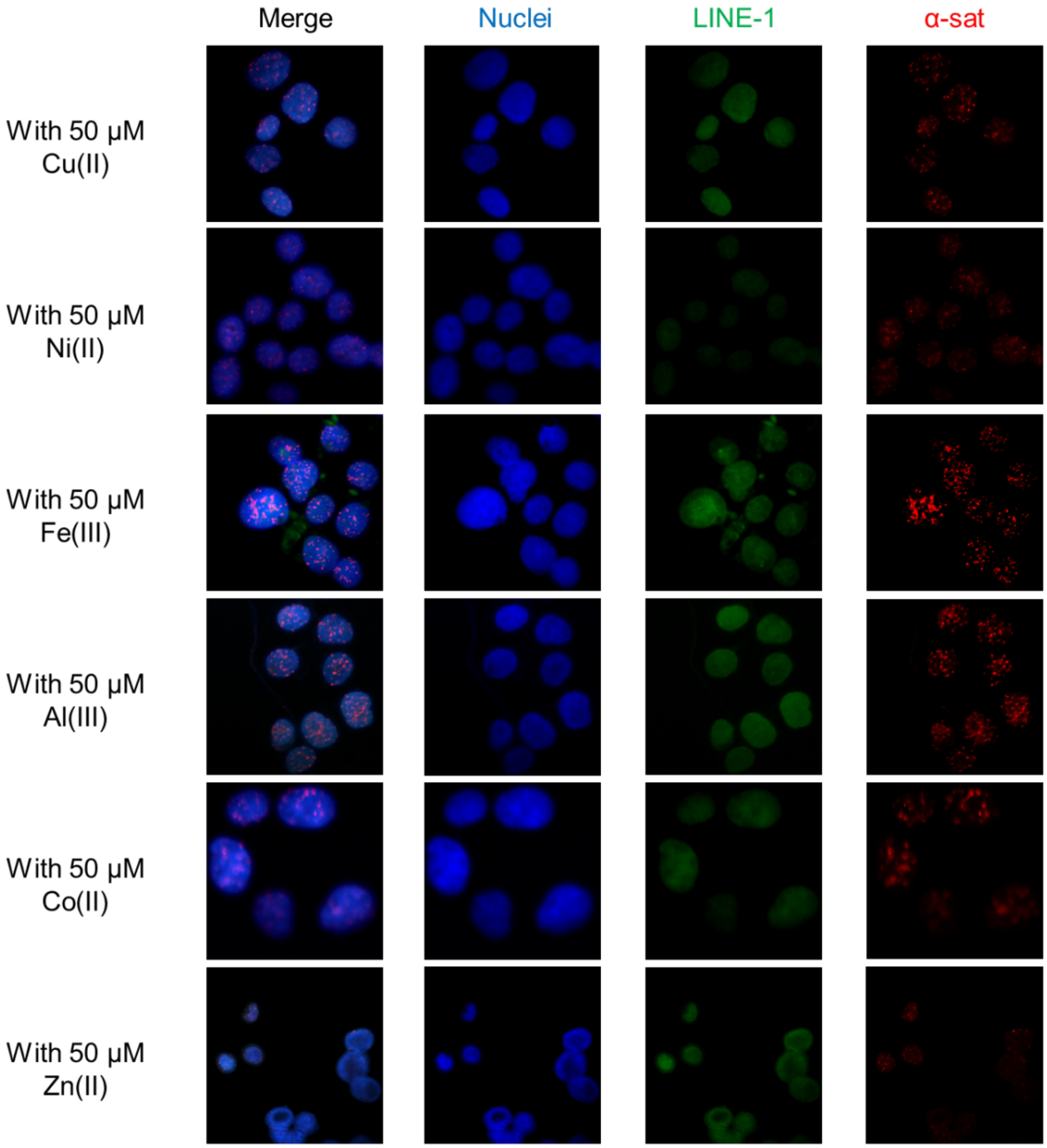

Supplementary Figure 2. MeFISH images of LINE-1 (green) and α-sat (red) from HepG2 cells treated with Cu(II), Ni(II), Fe(III), Al(III), Co(II) and Zn(II), ranging from 50 μM to 200 μM.

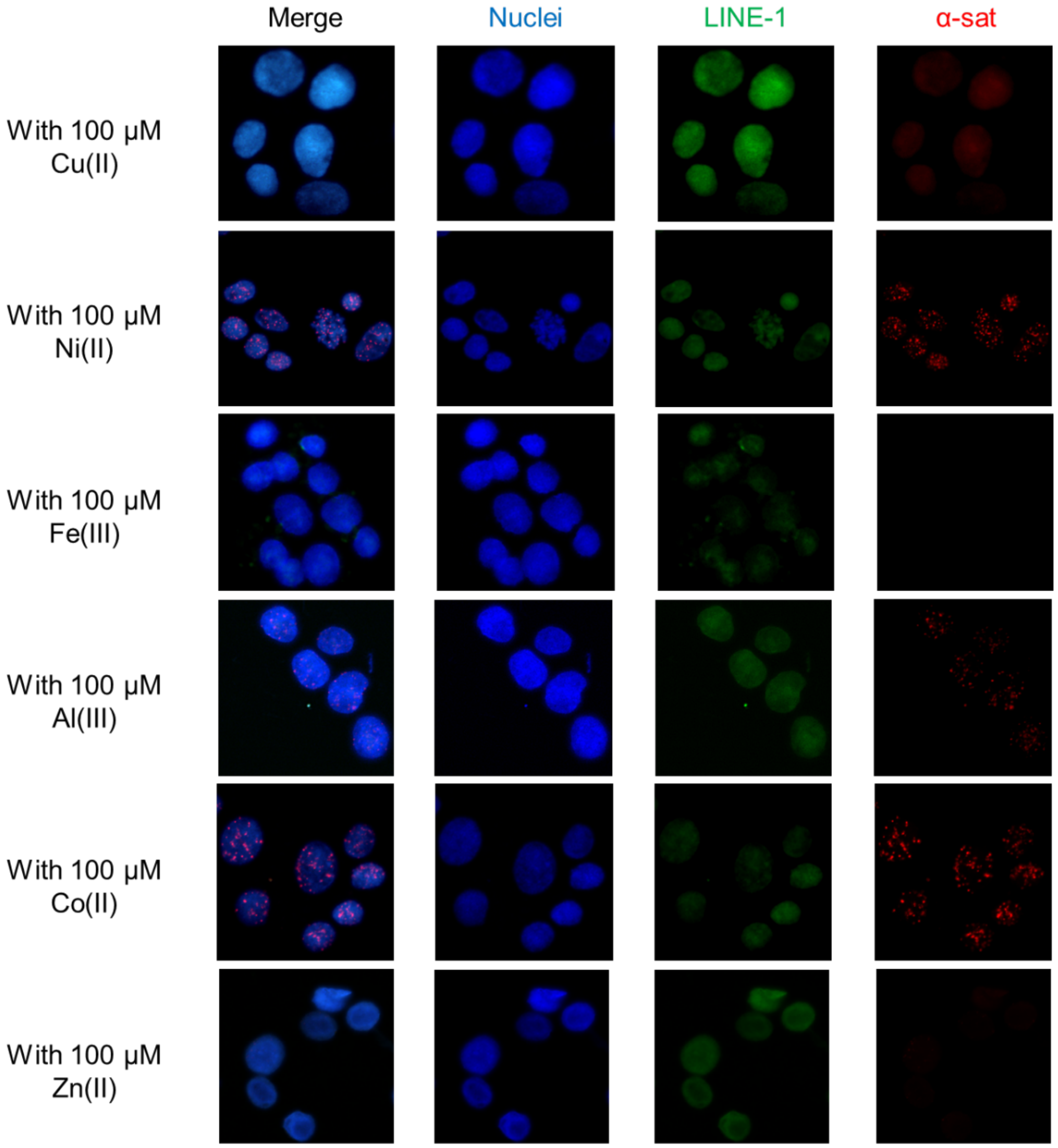

Supplementary Figure 1 (continued).

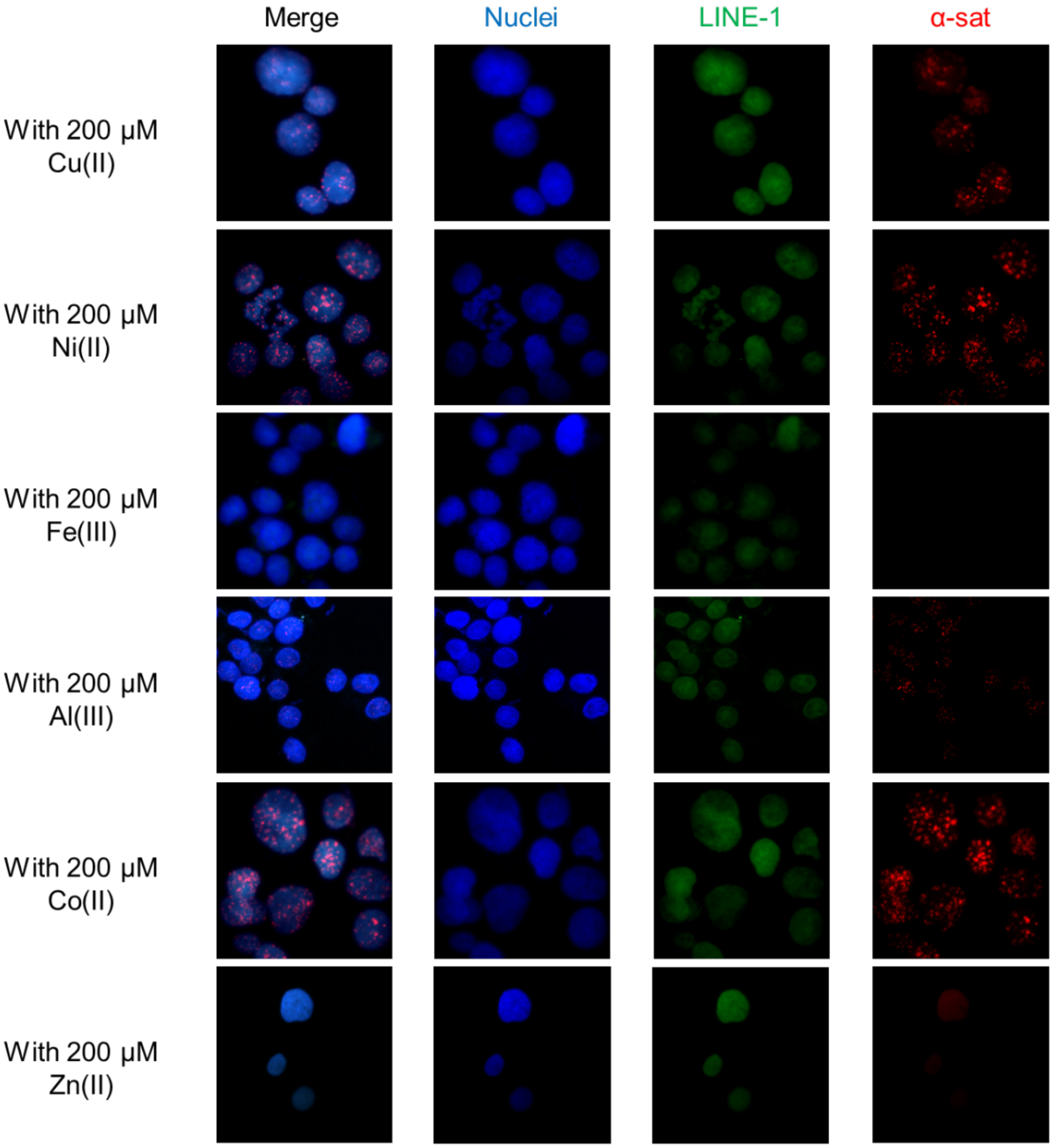

Supplementary Figure 2 (continued).

Supplementary Table 1. Cell viability assay. The relative cell viability was expressed as the value relative to the blank control cells.

Concentration /μM	Cu(II)	Ni(II)	Al(III)	Fe(III)	Co(II)	Zn(II)
**20**	1.11	1.03	0.86	1.17	1.21	0.92
**50**	1.08	0.97	1.12	1.15	1.12	0.91
**100**	1.02	1.04	1.16	1.13	1.18	0.98
**150**	0.85	1.10	1.14	1.08	1.27	0.87
**200**	0.93	1.03	1.14	0.91	1.15	1.01

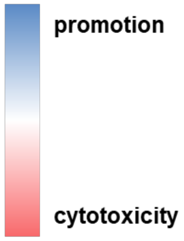

Supplement: Supplementary file 1 [file DataSheet1.pdf]
